# Shen-Yuan-Dan Capsule Attenuates Atherosclerosis and Foam Cell Formation by Enhancing Autophagy and Inhibiting the PI3K/Akt/mTORC1 Signaling Pathway

**DOI:** 10.3389/fphar.2019.00603

**Published:** 2019-05-31

**Authors:** Mingxue Zhou, Pan Ren, Ying Zhang, Sinai Li, Mengjie Li, Ping Li, Juju Shang, Weihong Liu, Hongxu Liu

**Affiliations:** ^1^Beijing Hospital of Traditional Chinese Medicine, Capital Medical University, Beijing Institute of Traditional Chinese Medicine, Beijing, China; ^2^Department of Traditional Chinese Medicine, Beijing Luhe Hospital, Capital Medical University, Beijing, China; ^3^Beijing Hospital of Traditional Chinese Medicine, Capital Medical University, Beijing, China

**Keywords:** atherosclerosis, foam cells, autophagy, PI3K/Akt/mTORC1, inflammation, Shen-Yuan-Dan Capsule

## Abstract

**Background and Aim:** The phosphoinositide 3-kinase (PI3K)/Akt/mammalian target of rapamycin complex 1 (mTORC1) signaling pathway plays a crucial role in autophagy and inflammation. Our previous studies demonstrated that Shen-Yuan-Dan Capsule (SYDC), a Chinese medicine used for treating angina pectoris, has anti-atherosclerotic and anti-inflammatory effects in mice. However, its effects on autophagy and the PI3K/Akt/mTORC1 signaling pathway remain unclear. This study aimed to explore the effects of SYDC on autophagy and PI3K/Akt/mTORC1 signaling in the apolipoprotein E knockout (ApoE^−/−^) mouse model and in macrophage-derived foam cells to delineate the underlying mechanism.

**Methods:** After 6 weeks of high-fat diet, ApoE^–/–^ mice were randomly grouped into control, Lipitor, low-SYDC (SYDC-L), middle-SYDC (SYDC-M), and high-SYDC (SYDC-H) groups (n = 10). The mice were intragastrically administered the respective treatment for 6 weeks. Murine RAW264.7 cells were stimulated with oxidized low-density lipoprotein (ox-LDL) (80 µg/ml) for 24 h and then pretreated with SYDC freeze-dried powder for another 24 h. Cells treated with SYDC were co-cultured for 24 h with LY294002, tricirbine, and rapamycin to investigate the effects on the PI3K/Akt/mTORC1 signaling pathway.

**Results:** SYDC ameliorated blood lipid levels, reduced the atherosclerotic index and plaque areas in the aortic root in mice, and inhibited total cholesterol (TC) levels and cholinesterase (ChE)/TC ratios in ox-LDL stimulated macrophages. Moreover, SYDC up-regulated Beclin1 and LC3II/I proteins in mice and in the ox-LDL–stimulated macrophages. Moreover, SYDC inhibited AKT phosphorylation at Ser473 and mTOR phosphorylation at Ser2448 in mice and in ox-LDL–stimulated macrophages. Furthermore, SYDC’s inhibitory of ChE/TC ratios in ox-LDL–stimulated macrophages was not changed by selective inhibition of the PI3K/Akt/mTORC1 pathway.

**Conclusions:** Our results highlight that SYDC treatment attenuates foam cell formation by promoting autophagy *via* inhibiting activation of the PI3K/Akt/mTORC1 signaling pathway. This study provides new insights into the molecular mechanism underlying SYDC’s therapeutic potential for treating atherosclerosis.

## Introduction

A growing body of evidence suggests that atherosclerosis and its related cardiovascular diseases are the leading cause of death and morbidity in developed countries (Wu et al., 2009; Getz and Reardon, 2011; Fu et al., 2014). Atherosclerosis is a complex disease that is caused by multiple factors, including lipid deposition, inflammation, and foam cells formation (Ross, 1999), the latter of which has been implicated as a key mediator of atherosclerosis development (Yuan et al., 2012). Subendothelial accumulation of lipid laden macrophages derived foam cells occurs during the early stage of atherosclerosis (Allahverdian et al., 2012). Accumulation of cholesterol esters in macrophages, as a hallmark of foam cell formation, depends on the uptake of oxidized low-density lipoprotein (ox-LDL) (Hansson, 2005). Ox-LDL stimulates macrophages to release pro-inflammatory cytokines, such as interleukin (IL)-1β and tumor necrosis factor-α (TNF-α) to trigger pro-inflammatory and pro-oxidant events in the initiation, propagation, and activation of atherosclerosis (Steinberg, 1997; Kirii et al., 2003; Robbesyn et al., 2004). Therefore, inhibiting macrophage foam cell formation could be an effective approach to attenuate atherosclerosis.

Recently, autophagy, a compensatory and self-protecting catabolic cellular pathway to maintain cell homeostasis, has recently been implicated as a protective mechanism during atherosclerosis (Martinet and De Meyer, 2009). Blocking ox-LDL-stimulated macrophage-derived foam cell formation significantly attenuates atherosclerotic development *via* autophagic regulation (Li et al., 2004; Moore and Tabas, 2011). Autophagy was considered to be impaired during atherosclerotic development by regulating the dysfunction of lipid metabolism and inflammatory reaction (Abderrazak et al., 2015; De Meyer et al., 2015; Li et al., 2016a). Atherosclerosis research has shown that autophagy regulates cholesterol efflux in macrophages to affect formation of foam cells, In addition, autophagy deficiency leads to inflammasome hyperactivation (Razani et al., 2012), whereas moderate activation of autophagy can effectively inhibit atherosclerosis (Vindis, 2015). Therefore, promoting autophagy could be a potential strategy to attenuate atherosclerosis that has been treated as a potential therapeutic target for atherosclerosis (Li et al., 2016b; He et al., 2017).

The phosphoinositide 3-kinase (PI3K)/Akt/mammalian target of rapamycin complex 1 (mTORC1) pathway is the main signaling pathway in autophagy. PI3K phosphorylates Akt, resulting in the activation of mTORC1 and the subsequent inhibition of autophagy. Knockdown of mTORC1 has been shown to ameliorate dysregulated blood lipid metabolism and decrease plaque area (Wang et al., 2013). Furthermore, mTORC1 knockdown increased autophagy-related gene 13 (Atg13) dephosphorylation, which is an index of increased autophagy (Wang et al., 2013). Therefore, inhibiting activation of the PI3K/Akt/mTORC1 signaling pathway could be an effective approach to enhance autophagy.

Selective inhibition of the PI3K/Akt/mTOR signaling pathway has been shown to regulate macrophage autophagy and markedly affect atherosclerotic plaque inflammation, burden, and vulnerability (Zhai et al., 2014). Moreover, mTOR enhances foam cell formation (Wang et al., 2014) and, inhibiting mTOR, enhances ox-LDL-induced autophagy *in vitro* and restricts atherosclerosis in ApoE^−/−^ mice (Peng et al., 2014). mTOR inhibitor rapamycin had an effect of inhibition of plaque inflammation, independent of serum lipid levels (Chen et al., 2009). Therefore, inducing autophagy *via* inhibiting activation of the PI3K/Akt/mTORC1 signaling pathway plays a key role in ox-LDL-induced macrophage-derived foam cell formation.

Shen-Yuan-Dan Capsule (SYDC) is traditional Chinese medicine (TCM) compound that has been effectively used to treat coronary heart disease and angina pectoris (Liu et al., 1999; Shang et al., 2006). In addition, SYDC can inhibit lipid peroxidation during myocardial ischemia/reperfusion in rats, eliminate oxygen-free radicals in ischemic myocardium, and act as an anti-oxidant in myocardial tissue (Wen et al., 2010; Liu et al., 2011; Shang et al., 2011). Previous studies have shown that SYDC’s anti-atherosclerotic effects are mediated by inhibiting TNF-α in apolipoprotein E knockout (ApoE^−/−^) mice fed a high-fat diet (Zhou et al., 2017). However, the precise mechanism underlying SYDC’s anti-atherosclerotic properties remains to be elucidated.

Currently, the effects of SYDC on foam cell formation, autophagy, and PI3K/Akt/mTORC1 signaling have not been investigated. According to the close relationships of autophagy, PI3K/Akt/mTORC1 signaling, foam cell formation and inflammation, we hypothesized that SYDC protect macrophages from ox-LDL-induced foam cell formation by enhancing autophagy and regulating the PI3K/Akt/mTORC1 signaling pathway. Therefore, we evaluated the role of SYDC in protecting against foam cell formation and investigated the underlying mechanism of this protective effect.

## Materials and Methods

### Animals

Male ApoE^−/−^ mice (n = 50; 8 weeks of age; weight, 18-20 g) on the C57BL/6J background were purchased from Beijing Weitong LiHua Experimental Technology Co. Ltd (Beijing, China).

### Ethics Approval

All animal research conformed to the Guidelines for the Care and Use of Laboratory Animals published by the US National Institutes of Health (NIH publication no. 85-23) and was approved by the ethics review board for Animal Studies of Peking University Health Science Center (permit number: IMM-GuYC-1).

### Reagents

Kits for assessing total cholesterol (TC, 7007210506), triglycerides (TG, 7006210506), low-density lipoprotein cholesterol (LDL-C, 7021220605), and high-density lipoprotein cholesterol (HDL-C, 7020210506) in mouse serum were purchased from Yingkexinchuang Science and Technology Ltd. (Xiamen, China). Antibodies against Beclin1 (ab62557, ab207612), LC3II protein kinase B (ab48394, ab192890), pan-Akt, (ab8805), PI3K (ab182651), anti-phosphorylated (p)-PI3Kp85 (ab182651), anti-p-AKT1 (S473) (ab18206) mTOR (ab32028), p-mTORC1 (Ser2448) (ab109268), and Atg13 (ab105392) were purchased from Abcam (Cambridge, UK). LY294002 (HY-10108), Triciribine (HY-15457), and Rapamycin (HY-10219) were purchased from Med Chem Express (New Jersey, USA). Kits for assessing TC and free cholesterol (FC) (E1015 and E1016) in macrophages were purchased from Applygen Technologies Inc. (Beijing, China). SYDC was provided by the manufacturing laboratory of Beijing Tradition Chinese Medicine Hospital (Beijing, China; Z20053327). Lipitor (atorvastatin calcium) was purchased from Pfizer Pharmaceutical Co., Ltd (Shanghai, China; H20051408).

### UPLC–MS/MS Analysis of SYDC

We used a Waters ultra-high-performance liquid chromatography tandem mass spectrometry (UPLC–MS/MS) spectrometer equipped with a HESI-II probe to analyze SYDC. The positive and negative HESI-II spray voltages were 3.7 and 3.5 kV, respectively, and the heated vaporizer temperature was maintained at 320°C. The collision gas was nitrogen at a pressure of 1.5 mTorr. Acquity UPLC HSST3 column (1.8 μm × 2.1 mm × 100 mm) was selected for analyses. The mobile phase was composed of A (water, 0.1% formic acid, v/v) and B (methanol, 0.1% formic acid, v/v), with a linear gradient elution. The flow rate was set to 0.3 ml/min, and the column temperature was 45°C. Data were collected and processed using the Waters Masslynx 4.1 system.

### Establishment of the Atherosclerotic Model and Drug Treatment

All ApoE^−/−^ mice were fed a high-fat diet containing 21% (wt/wt) fat supplemented with 0.15% (wt/wt) cholesterol (Yang et al., 2013) purchased from Beijing Ke’aoXieli Feed Co. Ltd (Beijing, China) for 12 weeks. After 6 weeks of high-fat diet, ApoE^−/−^ mice were randomized into control, Lipitor (positive-control group, 3.34 mg/kg), high-SYDC (SYDC-H, 160 mg/kg), middle-SYDC (SYDC-M, 80 mg/kg), and low-SYDC (SYDC-L, 40 mg/kg) groups (n = 10). All mice were orally gavaged for 6 weeks in combination with high-fat diet feeding. High, middle, and low doses of SYDC were selected as twice the clinically relevant doses in humans, the clinically relevant dose, and half the clinically relevant dose in humans, respectively. The medical dose in mice was 9.01 times that used for humans (Zhou et al., 2008).

### Histology

After 6 weeks of treatment, all mice were euthanized with 0.1% pentobarbital sodium. Hearts were harvested from each mouse, and one third of the apical heart including the aortic sinus was fixed in 10% formaldehyde, embedded in paraffin, and sectioned to assess the morphology of atherosclerotic plaques using hematoxylin and eosin (H&E) staining. Aorta samples, except for the aortic root, were removed and stored at −80°C. Blood samples were collected from the left ventricle of male ApoE^−/−^ mice after 12 weeks of high-fat diet.

### Determination of Serum Lipid Concentration

Serum LDL-C and HDL-C levels were determined using immunoturbidimetry, and TC and TG levels were determined using enzymatic assays. All other indices were determined using the RX-2000 radiometer (Technicon Instruments Company, NY, USA). The atherosclerosis index (AI) was derived as AI = non-HDL-C/HDL-C (SK, 2008).

### Evaluation of Atherosclerotic Lesions and Composition

The morphology of atherosclerotic plaque was evaluated using H&E staining. A morphometric analysis was performed using Image-Pro Plus (Media Cybernetics, MD, USA). Sections (5 μm) (eight per group) from the same segment were quantified according to Suzuki et al. (1997). The atherosclerotic plaque area was measured directly and subtracted from the area enclosed by the internal elastic lamina to derive the patent lumen area (Johnson et al., 2006). Plaque area was normalized to the area surrounding the internal elastic lamina.

### Cell Culture and Treatment

RAW264.7 murine macrophages were obtained from the National Experimental Cell Resource Sharing platform (Beijing, China). Cells were cultured in Dulbecco’s modified Eagle medium (DMEM) supplemented with 10% fetal bovine serum (FBS) (Sigma, 1099-141) at 37°C in a humidified atmosphere containing 5% CO_2_ for 24 h. Media was then replaced with media lacking FBS and incubated for an additional 12 h. Cells (seeded at 1 × 10^5^ cells/ml) were treated with ox-LDL (80 μg/ml) with or without SYDC (1.5625, 3.125, and 6.25 mg/ml) for 24 h. Cells treated with phosphate-buffered saline (PBS) were used as the controls. Cells were co-cultured for 24 h with LY294002 (3.2 μmol/L), Tricirbine (0.4 μmol/L), and rapamycin (3.125 μmol/L) to investigate the role of the PI3K/Akt/mTORC1 pathway. Cells in each treatment group were collected and analyzed as described below.

SYDC lyophilized powder was prepared by the Institute of Traditional Chinese Medicine, Chinese Academy of Traditional Chinese Medicine. Cold trap temperature was −45°C; vacuum, −0.1. A 100-mg Shen-yuan-dan freeze-dried powder was weighed and then sufficiently dissolved in 4 ml DMEM. The drug solution was filtered and sterilized with a filter membrane, and stored at 4°C. The powder was diluted to the desired concentration prior to experimentation.

### Cell Viability Assays

Cell viability was determined using the CCK-8 assay according to the manufacturer’s instructions. Cells were seeded at 1 × 10^5^ cells/ml in 96-well plates and then incubated with CCK-8 reagent for 3 h at 37°C. Absorbance was measured at 450 nm and expressed as an arbitrary unit proportional to cell toxicity. For each of these experiments, at least three parallel measurements were performed.

### Oil Red O Staining

Cells were washed three times in PBS and fixed with Acetone formaldehyde fixative for 15 min and then subsequently washed three times in PBS. Cells were stained with ORO in isopropanol (Sigma-Aldrich) and water (3:2 by volume) for 15 min. They were then washed three times in PBS. Cells with red-stained lipid droplets were observed and photographed using an inverted microscope (Olympus, Japan).

### Measurement of Cholesterol in Macrophages

The TC and FC levels were measured according to the manufacturer’s instructions (please see “Reagents” section). Briefly, cells were incubated in lysis buffer for 10 min. After high-speed centrifugation, the absorbance of the supernatants was measured at 550 nm. The TC levels in macrophages were calculated based on a titrated standard curve. For each experiment, at least three parallel measurements were performed. Cholinesterase (ChE) levels were derived as ChE = TC − FC. The ChE/TC ratio was derived as = (TC − FC)/TC.

### Western Blot

Aortic samples (n = 5 mice per group) were harvested and stored at −80°C until protein extraction. Protein expression of Beclin-1, LC3II, PI3K, p-Akt, mTORC1, p-mTORC1, and Atg13 were detected using Western blot analysis as previously described (Zhou et al., 2011). Primary antibodies targeting Beclin1 (1:1,000), LC3II (1: 1,000), PI3K (1:1,000), p-Akt (1:1,000), mTORC1 (1:2,000), p-mTORC1 (1:1,000), Atg13 (1:1,000), and glyceraldehyde-3-phosphate dehydrogenase (GAPDH) (1:1,000) were used for Western blot analysis. Primary antibody detection was performed using an enhanced chemiluminescence detection system (Vigorous, Beijing, China).

### Statistical Analysis

Data are presented as mean ± standard deviation. All statistical analyses were performed using SPSS 13.0. Normally distributed data were analyzed using one-way analysis of variance (ANOVA) with a Bonferroni *post hoc* test to evaluate the statistical significance of intergroup differences in all of the tested variables. A *P* value < 0.05 was considered to indicate statistical significance.

## Results

### SYDC Composition

We used UPLC–MS/MS to determine the composition of SYDC. As shown in **Supplementary Figure 1**, the total ion chromatograms of SYDC included Tetrahydropalmatine, Harpagoside, Salvianic acid A, Salvianolic acid B, and Tanshinone IIA. The mass spectrograms and chemical formulas of the main ingredients of SYDC are shown in **Supplementary Figures 2** and **3**.

### SYDC Inhibits Atherosclerosis in ApoE^−/−^ Mice

**Figure 1:**
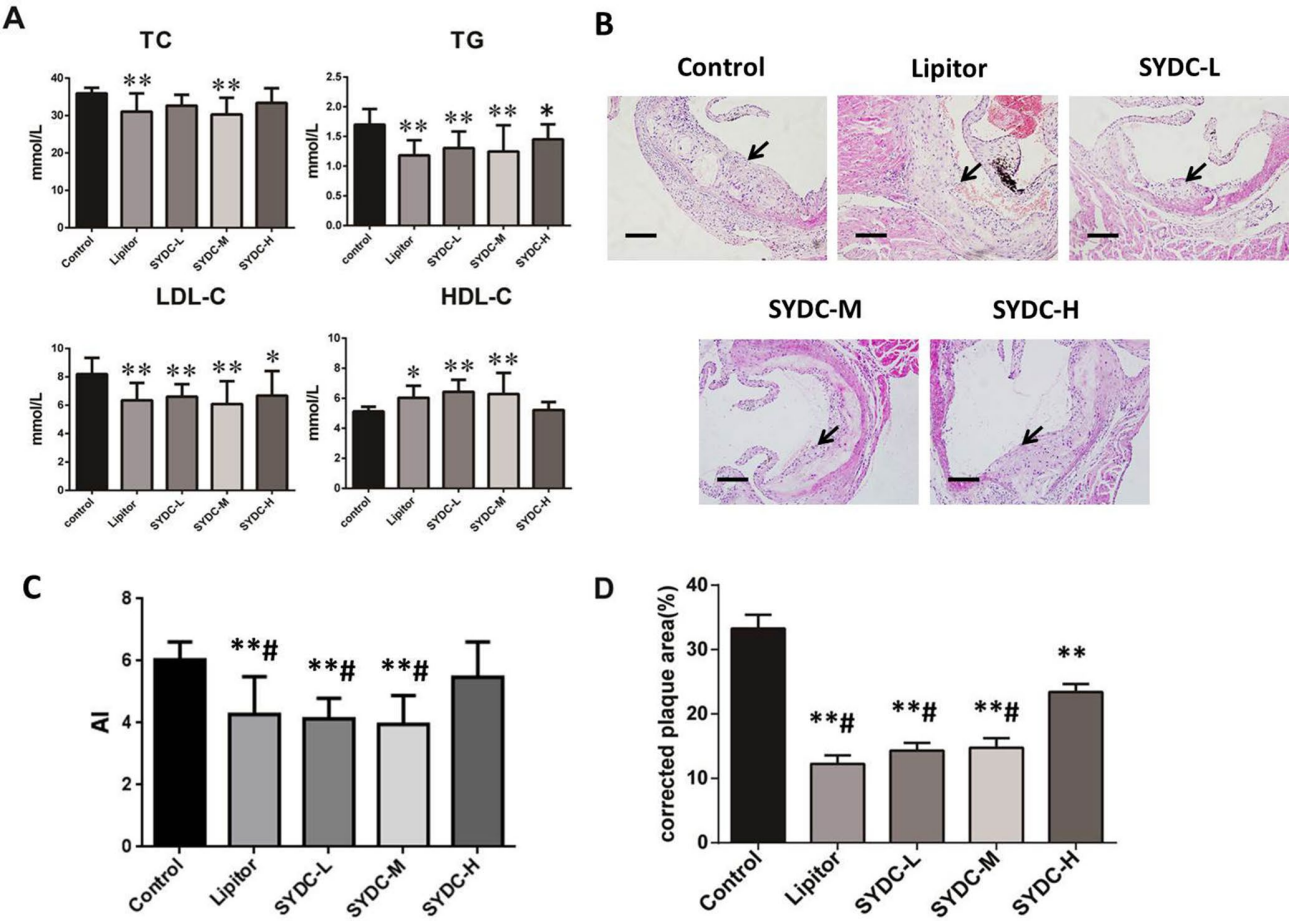
Effects of Shen-Yuan-Dan Capsule (SYDC) on atherosclerosis. **(A)** Blood lipid total cholesterol (TC), triglycerides (TG), low-density lipoprotein cholesterol (LDL-C), and high-density lipoprotein cholesterol (HDL-C) levels in the control, Lipitor, SYDC-L, SYDC-M, and SYDC-H groups (n = 10). **(B)** Representative images of H&E staining. Black arrows indicate atherosclerotic plaques. Tissues were examined using light microscopy (scale bars = 100 µm). **(C)** AI values in the control, Lipitor, SYDC-L, SYDC-M, and SYDC-H groups (n = 10). **(D)** Quantitation of atherosclerotic plaques in the different groups (n = 8). *P < 0.05 vs. control group; **P < 0.01 vs. control group; ^#^ *P* < 0.01 vs. SYDC-H group.

As shown in **Figure 1A**, compared with the control group, serum TC levels in the SYDC-M group and Lipitor group significantly decreased (*P* < 0.01), serum TG and LDL-C levels in all of the drug-treatment groups significantly decreased (*P* < 0.05, *P* < 0.01, respectively), and the HDL-C level in the SYDC-L, SYDC-M and Lipitor groups significantly increased (*P* < 0.05, *P* < 0.01, respectively). As shown in **Figure 1C**, AI in the SYDC-L, SYDC-M and Lipitor groups were significantly decreased compare with the control group and SYDC-H group (*P* < 0.01), but there were no significant differences observed in blood lipid indexes and AS between the SYDC-L,M groups and the Lipitor (positive control)group (*P* > 0.05)

After 6 weeks of drug administration, H&E staining and Image-pro plus analysis showed that the aortic plaque areas in the SYDC-L, SYDC-M, SYDC-H, and Lipitor groups significantly decreased compared to the control group (*P* < 0.01), and the aortic plaque areas in the SYDC-L, SYDC-M, and Lipitor groups significantly decreased compared to the SYDC-H group (*P* < 0.01) (**Figure 1B and D**).

### SYDC Promotes Autophagy in ApoE^−/−^ Mice

**Figure 2:**
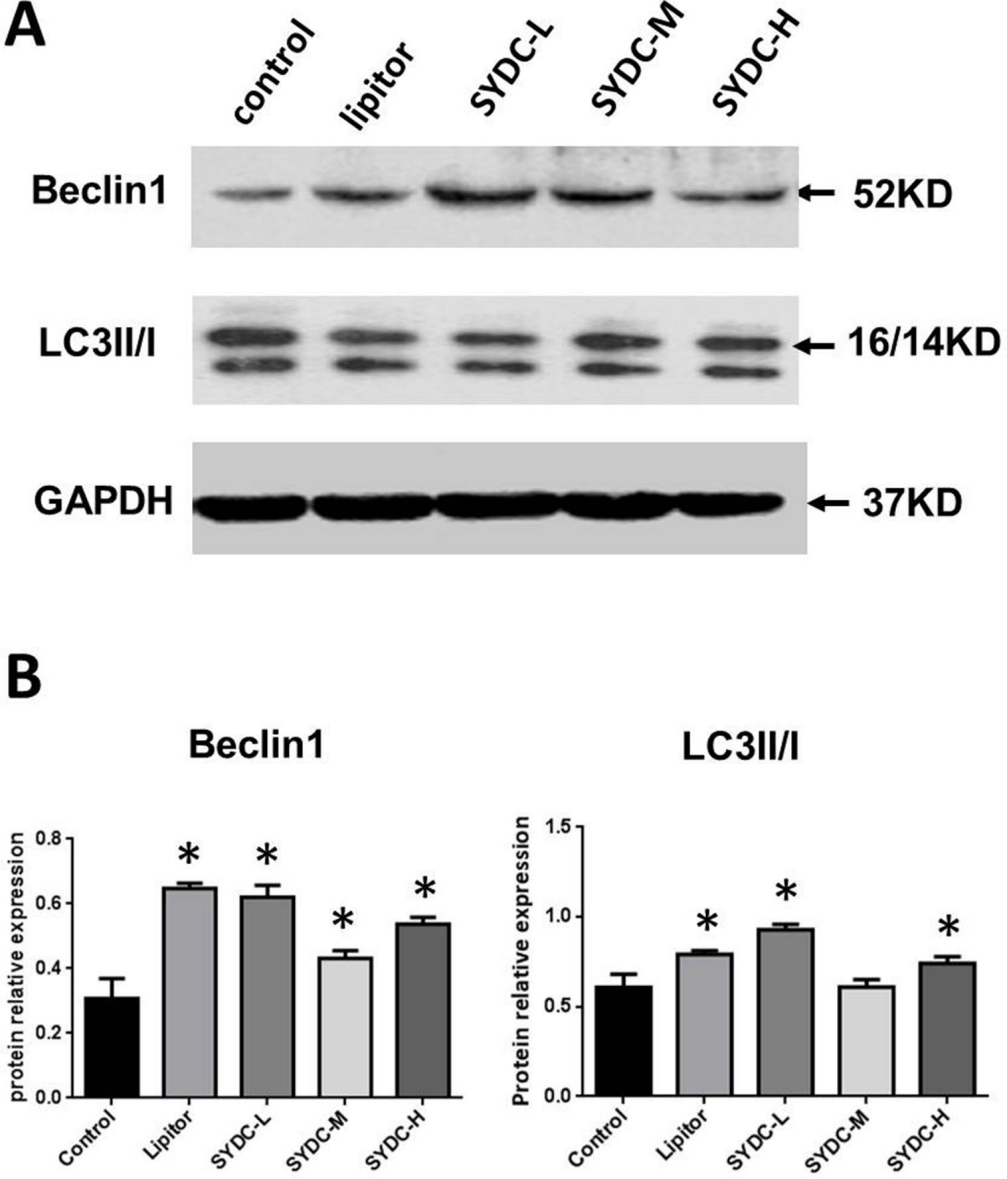
Effect of SYDC on autophagy. **(A)** Representative images of Western blot showing Beclin-1 expression and the LC3II/I ratio in aortic samples. GAPDH was used as a loading control. **(B)** Densitometry values of the western blot analysis were normalized to GAPDH expression and represented as relative intensity. *P < 0.01 vs. control group, SYDC-H, high-SYDC (160 mg/kg), SYDC-M, middle-SYDC (80 mg/kg), and SYDC-L, low-SYDC (40 mg/kg) (n = 5).

We next assessed Beclin1 expression and the LC3II/I ratios in the aortas of the atherosclerotic mice. As shown in **Figure 2A and B**, Beclin1 expression in the Lipitor, SYDC-L, SYDC-M, and SYDC-H groups and the LC3II/I ratios in the Lipitor, SYDC-L, and SYDC-H groups significantly increased compared to the control group (*P* < 0.01), but there were no significant differences in either Beclin1 or LC3II/I ratios between the drug-treatment groups (*P* > 0.01).

### SYDC Inhibits Activation of the PI3K/Akt/mTORC1 Signaling Pathway in ApoE^−/−^ Mice

**Figure 3:**
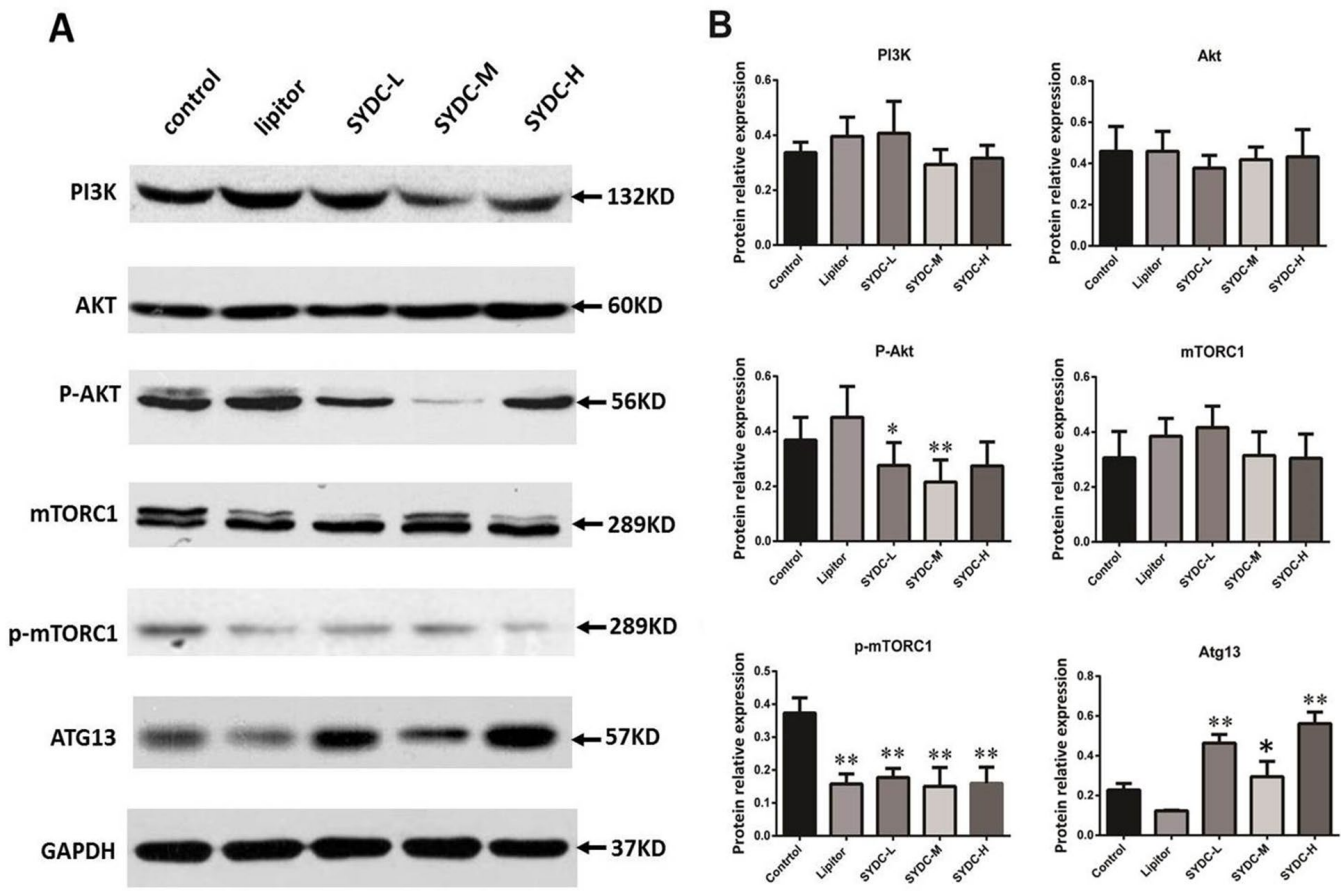
Effect of SYDC on the phosphoinositide 3-kinase (PI3K)/Akt/mammalian target of rapamycin complex 1 (mTORC1)/Atg13 signaling pathway. **(A)** Representative images of Western blot showing PI3K, p-Akt, mTORC1, p-mTORC1, and Atg13 expression in the aortas of the control, Lipitor, SYDC-L, SYDC-M, and SYDC-H groups. GAPDH was used as a loading control. **(B)** Densitometry values of the Western blot analysis were normalized to GAPDH expression and represented as relative intensity (n = 5). *P < 0.05 vs. control group; **P < 0.01 vs. control group.

The PI3K/Akt/mTORC1 signaling pathway and Atg13 are crucial for regulating autophagy. We next measured protein expression of PI3K, Akt, p-Akt, mTORC1, p-mTORC1, and Atg13 in the aortas of mice using Western blot analysis to determine the effect of SYDC on the PI3K/Akt/mTORC1/Atg13 signaling pathway. As shown in **Figure 3A and B**, there were no significant differences in protein expression of PI3K, Akt, and mTORC1 in the aortas among the five groups (*P* > 0.05). However, AKT phosphorylation at Ser473 and mTOR phosphorylation at Ser2448 in the SYDC groups were significantly reduced compared to the control (*P* < 0.05, *P* < 0.01), and protein expression of Atg13 in the SYDC-L, SYDC-M and SYDC-H groups was significantly increased (*P* < 0.05, *P* < 0.01).

### Effects of SYDC on the Viability of RAW264.7 Cells

**Figure 4:**
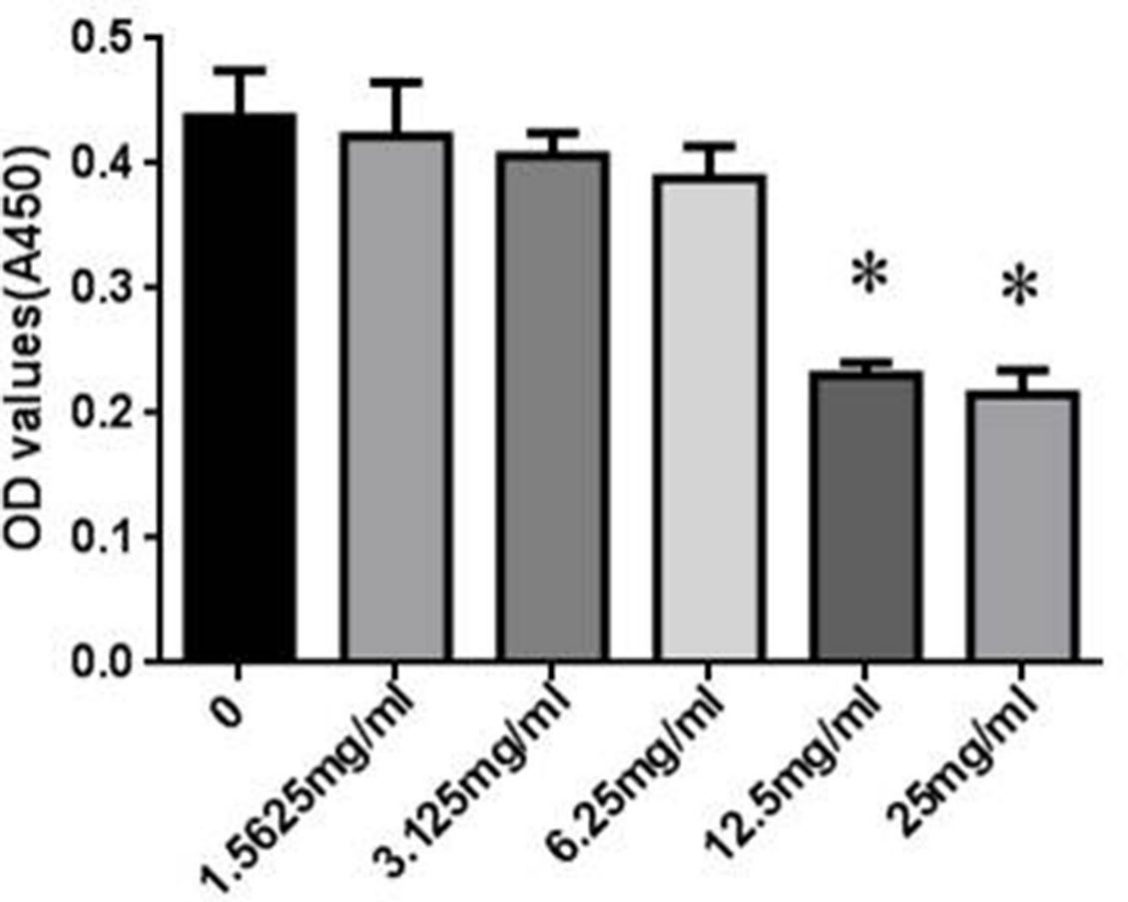
Effects of SYDC on the viability of RAW264.7 cells. RAW264.7 cells were treated with various concentrations of SYDC for 24 h, and cell viability was measured using the CCK- 8 assay. Data are expressed as the mean ± SD (n = 3), *P < 0.01 vs. control group alone.

The cytotoxicity of SYDC on RAW264.7 cells was assessed using the CCK-8 assay. RAW264.7 cells were incubated with different concentrations (0, 1.5625, 3.125, 6.25, 12.5, and 25 mg/ml) of SYDC for 24 h. As shown in **Figure 4**, SYDC significantly decreased cell viability at concentrations of 12.5 and 25 mg/ml (*P* < 0.01) while the viability of RAW264.7 cells was not affected by SYDC at concentrations below 6.25 mg/ml (*P* > 0.05). Therefore, all subsequent experiments used 1.5625, 3.125, and 6.25 mg/ml SYDC as the treatment concentration.

### SYDC Reduces TC Levels and the ChE/TC Ratio in ox-LDL Stimulated Macrophages

**Figure 5:**
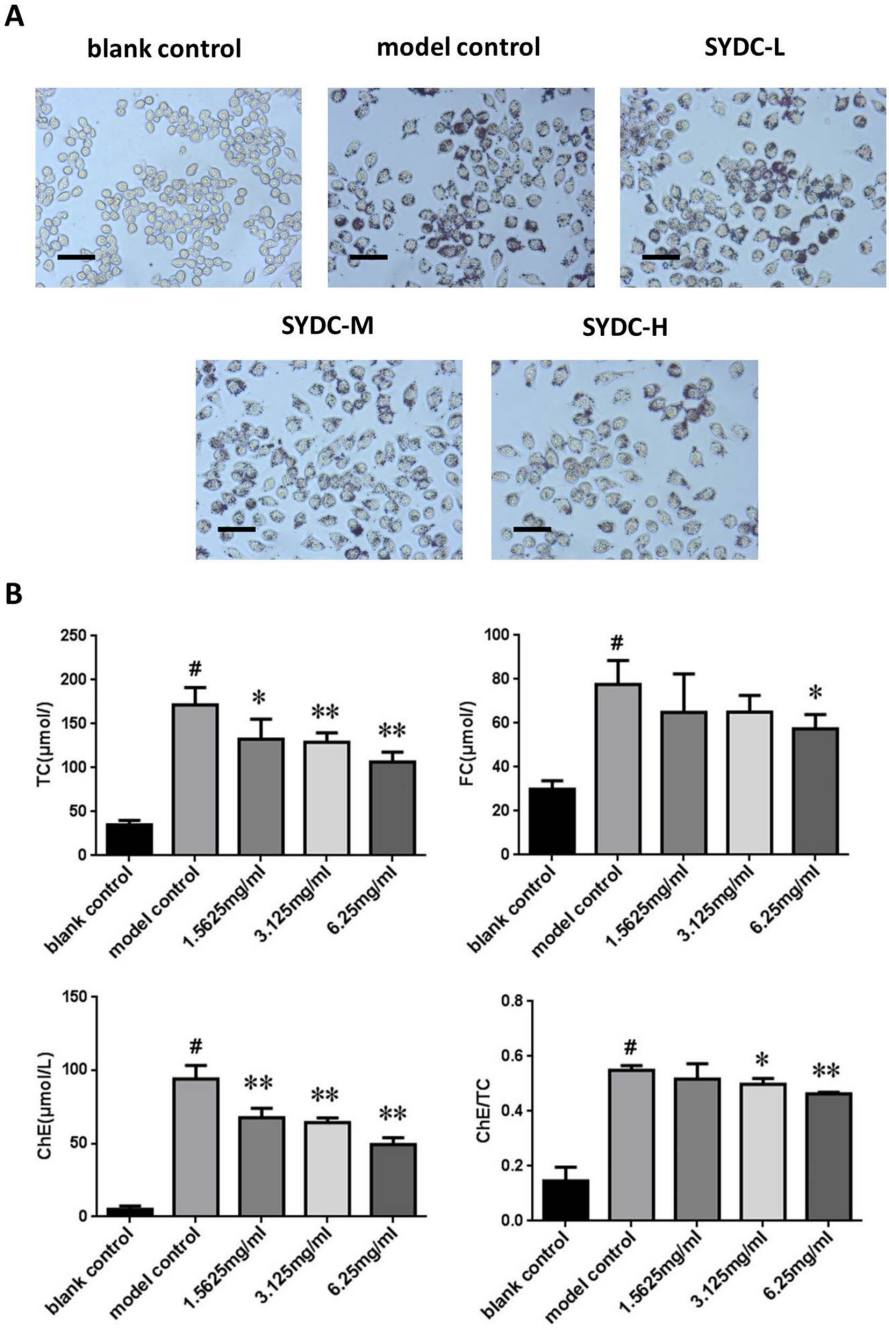
Effects of SYDC on the levels of total, free, and esterified cholesterol in ox-LDL-stimulated RAW264.7 cells. **(A)** Representative images of Oil red O-stained lipid droplets. Cells were examined by light microscope (Scale bars = 50 µm). **(B)** Effect of SYDC on the levels of total, free, and esterified cholesterol in ox-LDL-stimulated macrophages. The cells were treated with various concentrations of SYDC. TC, total cholesterol; FC, free cholesterol; ChE, cholesterol ester. Values are represented as mean ± SD, n = 3. ^#^ *P* < 0.01 vs. blank control group alone; *P < 0.05 vs. model control group alone, **P < 0.01 vs. model control group alone.

Macrophage transformation into foam cells is primarily stimulated by ox-LDL during atherosclerosis. To further assess the effect of SYDC on atherosclerosis, TC and FC levels were assessed *in vitro* using a cholesterol enzyme kit. As shown in **Figure 5**, TC, FC, and ChE levels and the ChE/TC ratio were significantly increased in the ox-LDL group compared to the control (*P* < 0.01). The TC and ChE levels in the ox-LDL after treatment with SYDC (1.5625, 3.125, and 6.25 mg/ml) were significantly reduced compared to the control group (*P* < 0.05, *P* < 0.01, respectively). The FC levels in the ox-LDL group after treatment with SYDC (6.25 mg/mL) were significantly reduced compared to the control group (*P* < 0.01). The ChE/TC ratio in the ox-LDL group after treatment with SYDC (3.125 and 6.25 mg/ml) was significantly reduced compared to the control group (*P* < 0.05, *P* < 0.01, respectively). In addition, Oil Red O staining and Image-pro plus analysis showed that the area of lipid drops in the ox-LDL group after treatment with SYDC (3.125 and 6.25 mg/ml) was significantly reduced compared to the control group (*P* < 0.05, *P* < 0.01, respectively). These data suggest that SYDC prevent formation of macrophage-derived foam cells and this inhibitory effect is dose-dependent.

### SYDC Promotes Autophagy in ox-LDL Stimulated Macrophages

**Figure 6:**
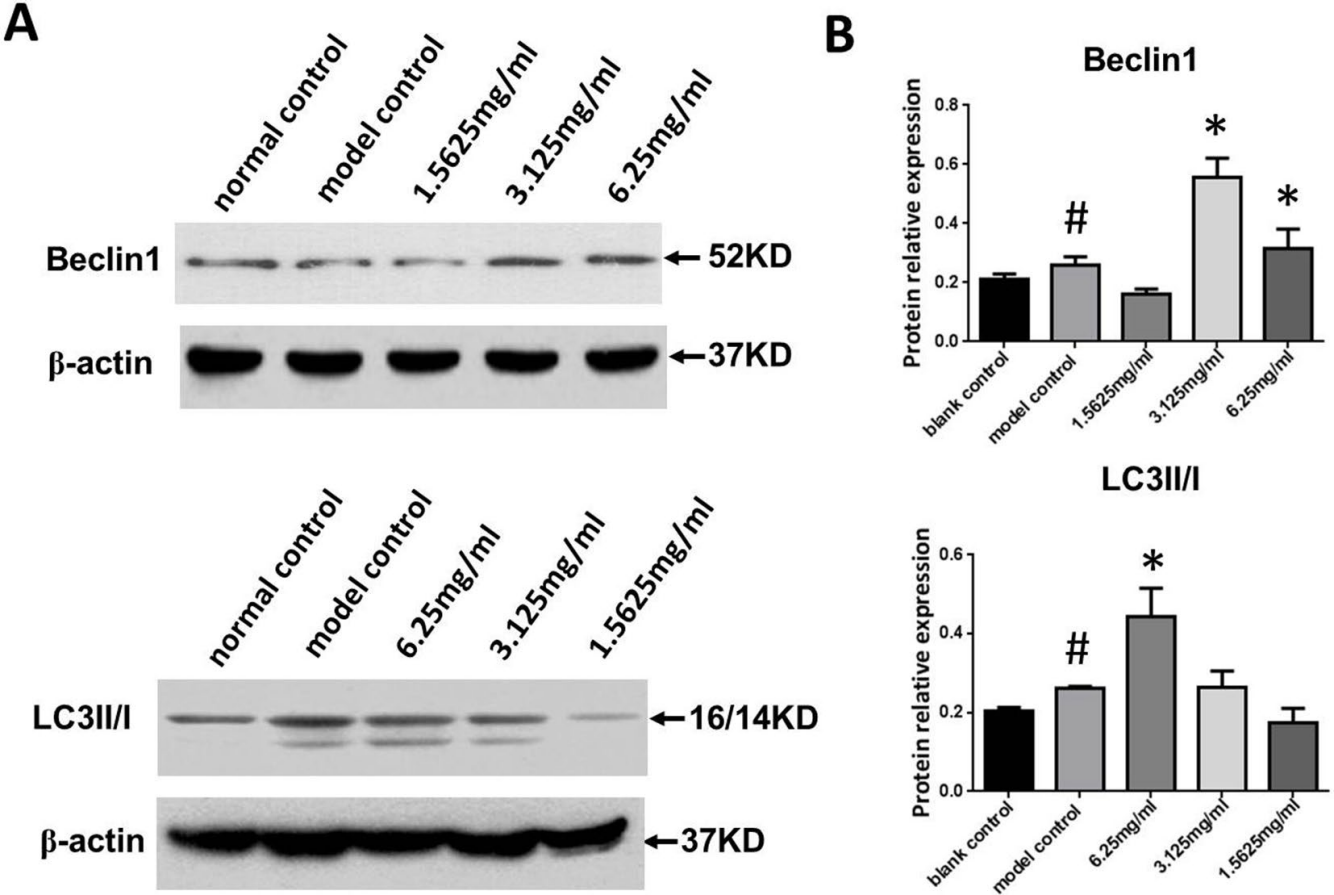
Effects of SYDC on autophagy. **(A)** Representative images of Western blot showing Beclin-1 expression and the LC3II/I ratio in RAW264.7 cells in the different groups. **(B)** Quantitation of Beclin-1 expression and LC3II/I ratio in RAW264.7 cells in different groups. GAPDH was used as a loading control. ^#^ *P* < 0.01 versus blank control group alone; *P < 0.01 vs. model control group alone, SYDC-H, high-SYDC (6.25 mg/ml), SYDC-M, middle-SYDC (3.125 mg/ml), and SYDC-L, low-SYDC (1.5625 mg/ml) (n = 3).

We next measured Beclin-1 expression and the LC3II/I ratio in ox-LDL stimulated macrophages. As shown in **Figure 6A and B**, Beclin-1 expression and the LC3II/I ratio in the ox-LDL group were significantly increased compared to the control group (*P* < 0.05, *P* < 0.01, respectively), and SYDC treatment (3.125 and 6.25 mg/mL) significantly increased Beclin1 expression and the LC3II/I ratio compared to the control group (*P* < 0.01).

### The Effect of SYDC on Activation of the PI3K/Akt/mTORC1 Signaling Pathway in ox-LDL Stimulated Macrophages

**Figure 7:**
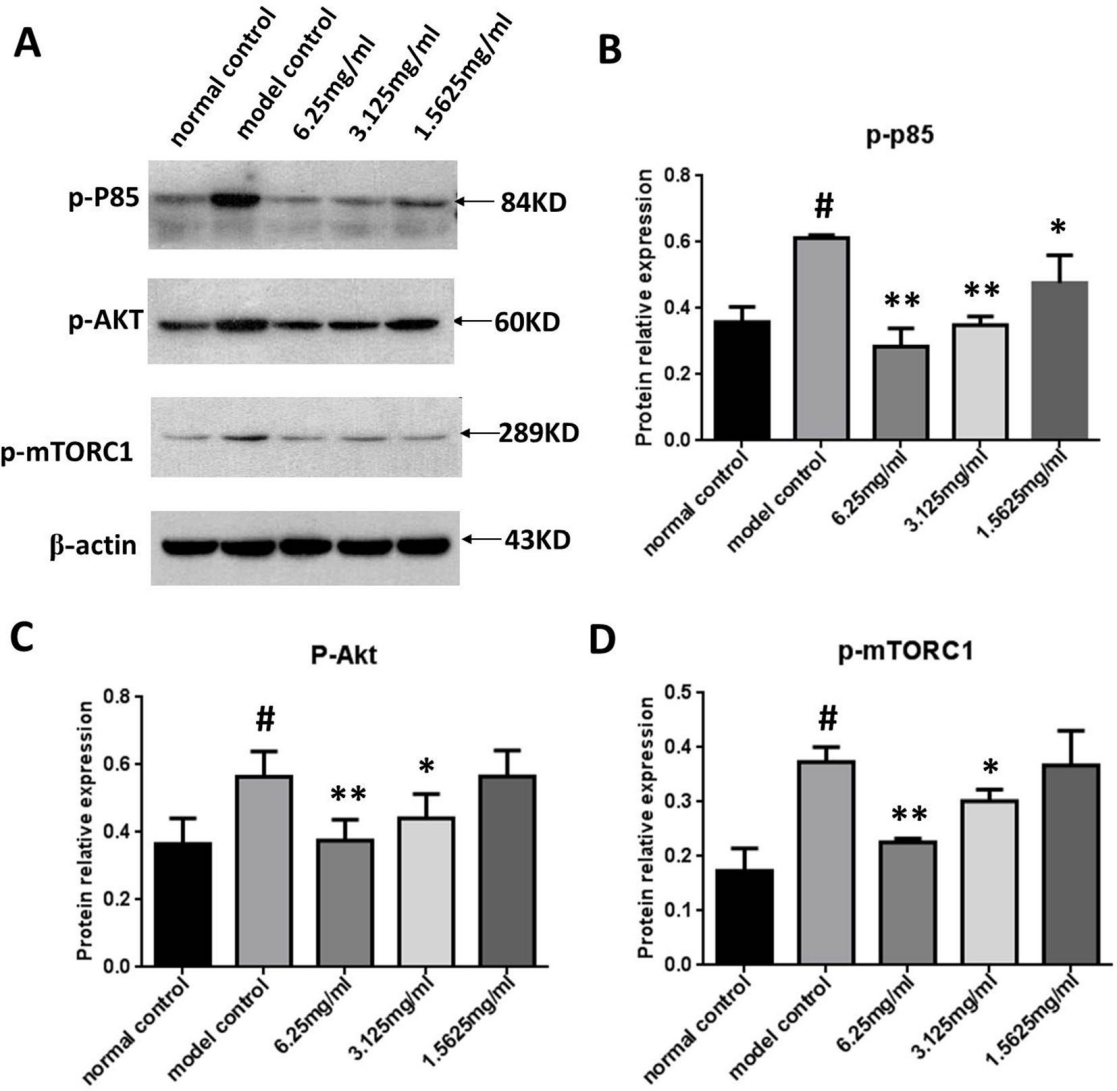
Effect of SYDC on the PI3K/Akt/mTORC1 signaling pathway in RAW264.7 cells. **(A)** Representative images of Western blot showing the p-PI3K, p-Akt, and p-mTORC1 expression in RAW264.7 cells in the different groups. GAPDH was used as a loading control. **(B)** Densitometry values of the Western blot analysis of p-p85 expression were normalized to GAPDH expression and represented as relative intensity (n = 5). **(C)** Densitometry values of the Western blot analysis of p-Akt were normalized to GAPDH expression and represented as relative intensity (n = 5). **(D)** Densitometry values of the Western blot analysis of p-mTORC1 were normalized to GAPDH expression and represented as relative intensity (n = 5). ^#^ *P* < 0.01 vs. blank control group alone; *P < 0.01 vs. model control group alone, SYDC-H, high-SYDC (6.25 mg/ml), SYDC-M, middle-SYDC (3.125 mg/ml), and SYDC-L, low-SYDC (1.5625 mg/ml) (n = 3).

We next measured p-PI3K, p-Akt, and p-mTORC1 expression in the ox-LDL stimulated macrophages using Western blot analysis to determine the effect of SYDC on the PI3K/Akt/mTORC1 signaling pathway. As shown in **Figure 7**, p-PI3K, p-Akt, and p-mTORC1 in the ox-LDL group were significantly increased compared to the control (all *P* < 0.01). SYDC (3.125 and 6.25 mg/ml) significantly decreased p-PI3K, p-Akt, and p-mTORC1 expressions compared to the control (*P* < 0.05, *P* < 0.01).

### SYDC Inhibits ox-LDL-Stimulated Macrophage Foam Cell Formation via the PI3K/Akt/mTORC1 Signaling Pathway

**Figure 8:**
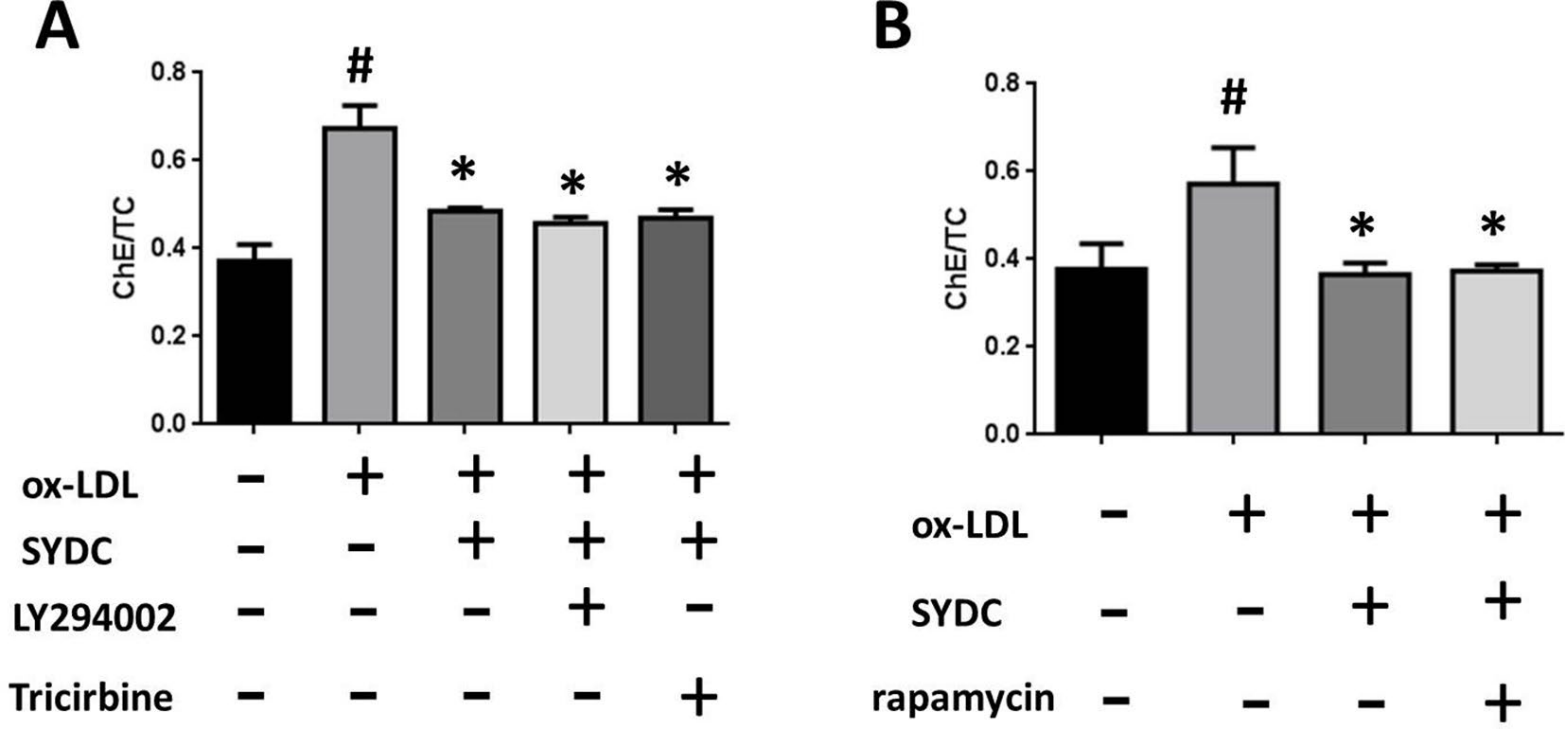
The effect of SYDC on the PI3K/Akt/mTOR1 signaling pathway on preventing macrophage foam cell formation. **(A)** Effect of the PI3K inhibitor LY294002 (3.2 µmol/L) and the Akt inhibitor Tricibin (0.4 µmol/L) on ChE/TC levels in ox-LDL-stimulated macrophages after SYDC treatment (6.25 mg/ml). **(B)** Effect of the mTORC1 inhibitor Ramamycin (3.125 µmol/L) on ChE/TC levels in ox-LDL-stimulated macrophages after SYDC treatment (6.25 mg/ml). Data are expressed as the mean ± SD, n = 3; ^#^ *P* < 0.01 vs. blank control group, *P < 0.01 vs. the model control group.

To determine the mechanism by which SYDC ameliorates atherosclerosis *via* the PI3K/Akt/mTORC1 signaling pathway, we tested the effects of LY294002 (PI3K inhibitor), TRICI (p-Akt inhibitor), and rapamycin (mTORC1 inhibitor) on ox-LDL-stimulated macrophages. As shown in **Figure 8A and B**, the ChE/TC ratios were significantly induced in the ox-LDL group compared to the control group (*P* < 0.01), and SYDC (6.25 mg/mL) significantly reversed this effect (*P* < 0.01). LY294002, TRICI, and rapamycin, did not significantly affect the ChE/TC ratios in the ox-LDL-group after SYDC treatment (*P* > 0.05), but the ChE/TC ratios in the ox-LDL-stimulated group following SYDC treatment were significantly decreased compared to the control group (*P* < 0.01).

## Discussion

Autophagy plays a crucial role in the development of atherosclerosis and is primarily regulated by the PI3K/Akt/mTORC1 signaling pathway. SYDC, a Chinese medicine used to treat angina pectoris, has been shown to have anti-atherosclerotic effects in mice models. However, its precise mechanism remains unclear. In the present study, we demonstrated that SYDC can inhibit atherosclerotic plaque development in ApoE^−/−^ mice and ameliorate macrophage lipid accumulation by enhancing autophagy. Moreover, we demonstrated that the PI3K/Akt/mTORC1 signaling pathway is involved in this process.

SYDC is a TCM compound that contains eight crude Chinese medicinal agents: *Salvia miltiorrhiza Bunge*, *Astragalus robustus* Bunge, *Codonopsis pilosula* (Franch). Nannf, *Scrophularia aestivalis* Griseb, leech, Lumbricus, Eupolyphaga Steleophaga, and *Corydalis tuberipisiformis* Z.Y.Su. Clinically, SYDC has been used to treat coronary heart disease and unstable angina pectoris (Liu et al., 1999). Atherosclerosis is the pathological basis of angina pectoris. Our previous study revealed that SYDC exerts an anti-atherosclerotic effect in ApoE^−/−^ mice fed a high-fat diet, and that the possible underlying mechanism involved inhibition of TNF-α (Zhou et al., 2017). Our current study suggests that a low and middle dose of SYDC have superior anti-atherosclerotic effect, which includes ameliorating blood lipids and reducing the AI and plaque areas in the aortic root of ApoE^−/−^ mice. Thus, SYDC can dose-independently decrease atherosclerosis *in vivo*.

The accumulation of lipid-containing macrophages in advanced atherosclerotic lesions induces an inflammatory response and enlargement of the lipid core, both of which contribute to the vulnerability of atherosclerotic lesions and the occurrence of acute cardiovascular diseases (Zhang et al., 2016). In addition, macrophage transformation into foam cells is primarily stimulated by ox-LDL, which plays a critical role in triggering proinflammatory events in the development of atherosclerosis. Our previous study revealed that SYDC exerts an anti-atherosclerotic effect in mice model by inhibiting inflammation (Zhou et al., 2017), but the effect of SYDC on lipid accumulation in macrophage is unclear. In this study, our data suggest that SYDC could prevent atherosclerosis by inhibiting foam cell formation stimulated by ox-LDL and that this inhibitory effect is dose-dependent. Many *in vivo* factors, including ox-LDL, inflammatory factors, and hematodynamics, affect the formation and development of atherosclerosis, while *in vitro* ox-LDL-stimulated macrophage-derived foam cell formation is only one important pathological link in the formation and development of atherosclerosis. Other pathological links, such as smooth muscle cell proliferation, also affect the formation and development of atherosclerosis. Thus, it is reasonable that there are dose-dependent differences of SYDC *in vivo* and *in vitro*. These data indicated that SYDC may exert anti-inflammatory effects to attenuate atherosclerosis by preventing macrophage lipid accumulation.

Accumulating evidence has indicated that autophagy could be used as a diagnostic and prognostic indicator of atherosclerosis (Razani et al., 2012; Zhao et al., 2013; Shao et al., 2016). The factors that induce atherosclerosis, such as ox-LDL, inflammation, and metabolic stress, can also stimulate autophagy (Zhao et al., 2013). Moderate activation of autophagy can effectively inhibit atherosclerosis by regulating cholesterol efflux in macrophages to inhibit formation of foam cells and inflammation reaction (Abderrazak et al., 2015; De Meyer et al., 2015; Li et al., 2016a). Beclin-1 is primarily responsible for autophagosome assembly and recruitment of other Atgs (Cuervo, 2006). LC3, a key autophagy protein marker, is converted from the cytoplasmic LC3-I form to the lipidated LC3-II form during autophagy. The LC3-II/I ratio is commonly used to monitor autophagy (Mizushima et al., 2010). In the present study, SYDC enhanced autophagy by up-regulating Beclin-1 expression and the LC3-II/I ratio in the aortas of atherosclerotic mice. In addition, we found that ox-LDL stimulation reduced autophagy, as evidenced by decreased Beclin-1 expression and LC3-II/I ratio, and that SYDC treatment rescued the autophagy pathway. Our findings indicate that SYDC can inhibit atherosclerotic plaque development in ApoE^−/−^ mice and ameliorate macrophage lipid accumulation by enhancing autophagy.

PI3K/Akt/mTORC1, a classic autophagic signaling pathway, has been reported to serve an important role in the regulation of autophagy in atherosclerosis (Zhai et al., 2014; Jiang et al., 2017). Selective inhibition of the PI3K/Akt/mTOR signaling pathway can regulate macrophage autophagy and markedly inhibit atherosclerotic plaque inflammation and foam cell formation (Wang et al., 2014; Zhai et al., 2014). Atg13 is an early initiator molecule in the autophagy pathway and is an important target of the PI3K/Akt/mTOR signaling pathway (Hosokawa et al., 2009; Mercer et al., 2009). The present study revealed that SYDC significantly decreased p-Akt and p-mTORC1, but increased Atg13 expression in ApoE^−/−^ mice. In addition, activation of PI3K, Akt, and mTORC1 was inhibited by SYDC treatment in ox-LDL-stimulated macrophages. Moreover, selective inhibition of PI3k, Akt, or mTORC1, respectively, did not enhance the effects of SYDC. These data suggest that the PI3K/Akt/mTORC1 signaling pathway effectively function following SYDC and inhibit the accumulation of lipids in macrophages by enhancing autophagy. Interestingly, our previous study also demonstrated that SYDC prevents the development of atherosclerosis and inflammatory reaction by inhibiting the activation of PI3K/Akt/NF-κB signaling pathway (Zhou et al., 2017), while another previous study also demonstrated that SYDC protects ischemic myocardium from ischemia-reperfusion injury and inhibits cell apoptosis in cardiomyocytes, and the mechanism underlying the cardioprotective effects is associated with activation of the PI3K/Akt pathway (Liu et al., 2013). Therefore, we speculate that PI3K/Akt may be a key signaling pathway for the cardiovascular protective effects of SYDC treatment.

## Conclusions

This is a novel study demonstrating that SYDC treatment attenuates foam cell formation by promoting autophagy and inhibiting activation of the PI3K/Akt/mTORC1 signaling pathway to prevent the development of atherosclerosis. Our findings provide new insights into the molecular mechanism of SYDC and its therapeutic potential to treatment atherosclerosis.

## Author Contributions

MZ and HL contributed to the conception and design of research. MZ, PR, SL, JS, ML and YZ performed the experiments. MZ, PR, SL, JS, YZ, WL, PL, and HL analyzed the data. MZ, PR, ML, and SL interpreted the results of the experiments. MZ and PR prepared the figures. MZ drafted the manuscript. HL edited and revised the manuscript.

## Funding

This work was supported by grants from the National Natural Science Foundation of China (grant 81673744) and the Beijing Natural Science Foundation (7162043).

## Conflicts of Interest Statement

The authors declare that the research was conducted in the absence of any commercial or financial relationships that could be construed as a potential conflict of interest.
