# Supplementary figures and images for: Shen-Yuan-Dan Capsule Attenuates Atherosclerosis and Foam Cell Formation by Enhancing Autophagy and Inhibiting the PI3K/Akt/mTORC1 Signaling Pathway
Source: Front Pharmacol. 2019 May 31;10:603. doi: 10.3389/fphar.2019.00603 (PMC6554665; doi:10.3389/fphar.2019.00603)

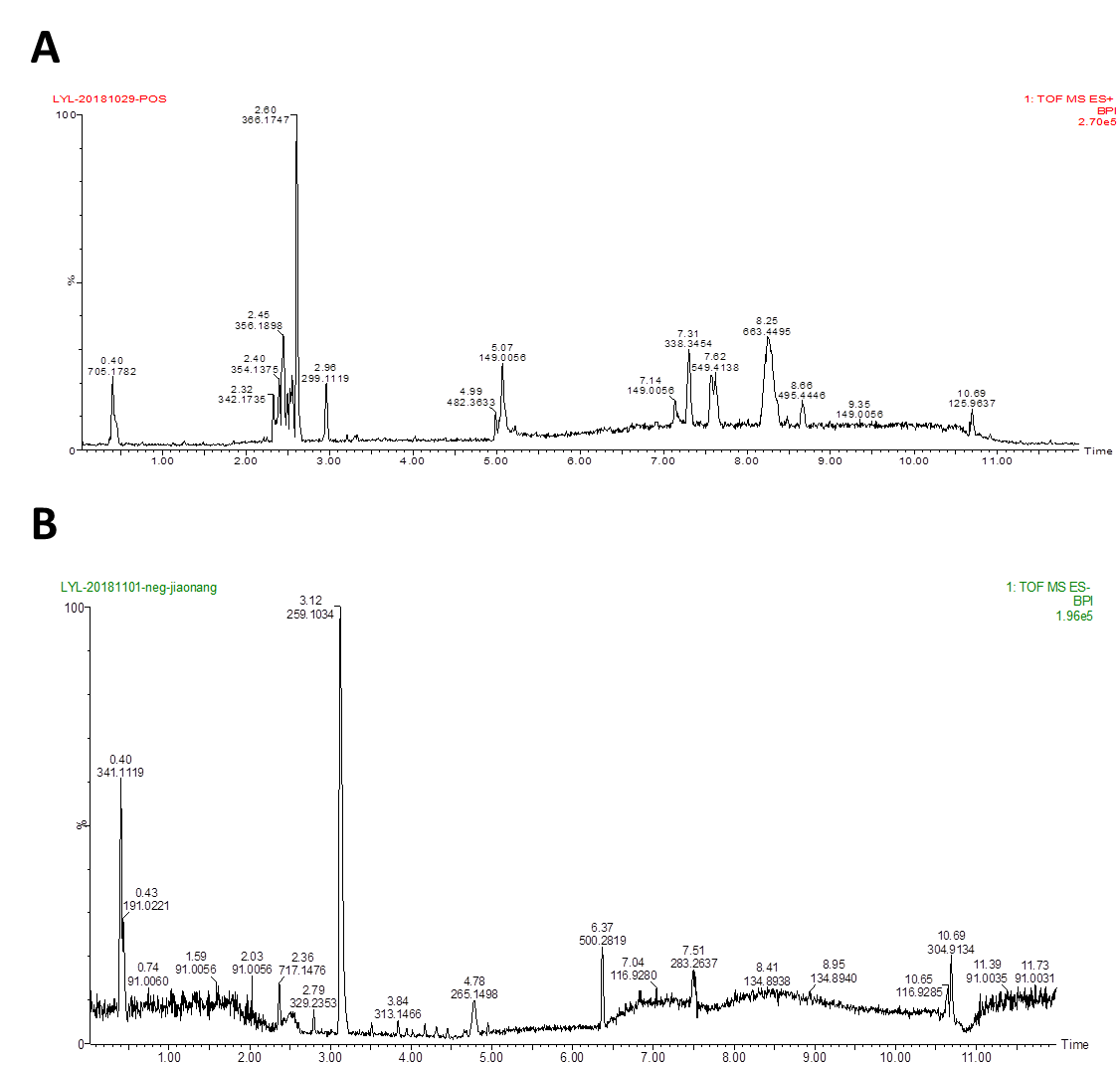

Supplement: Supplementary file 1 [file DataSheet_1.zip › Supplementary figures/Supplementary Figure 1.tif]

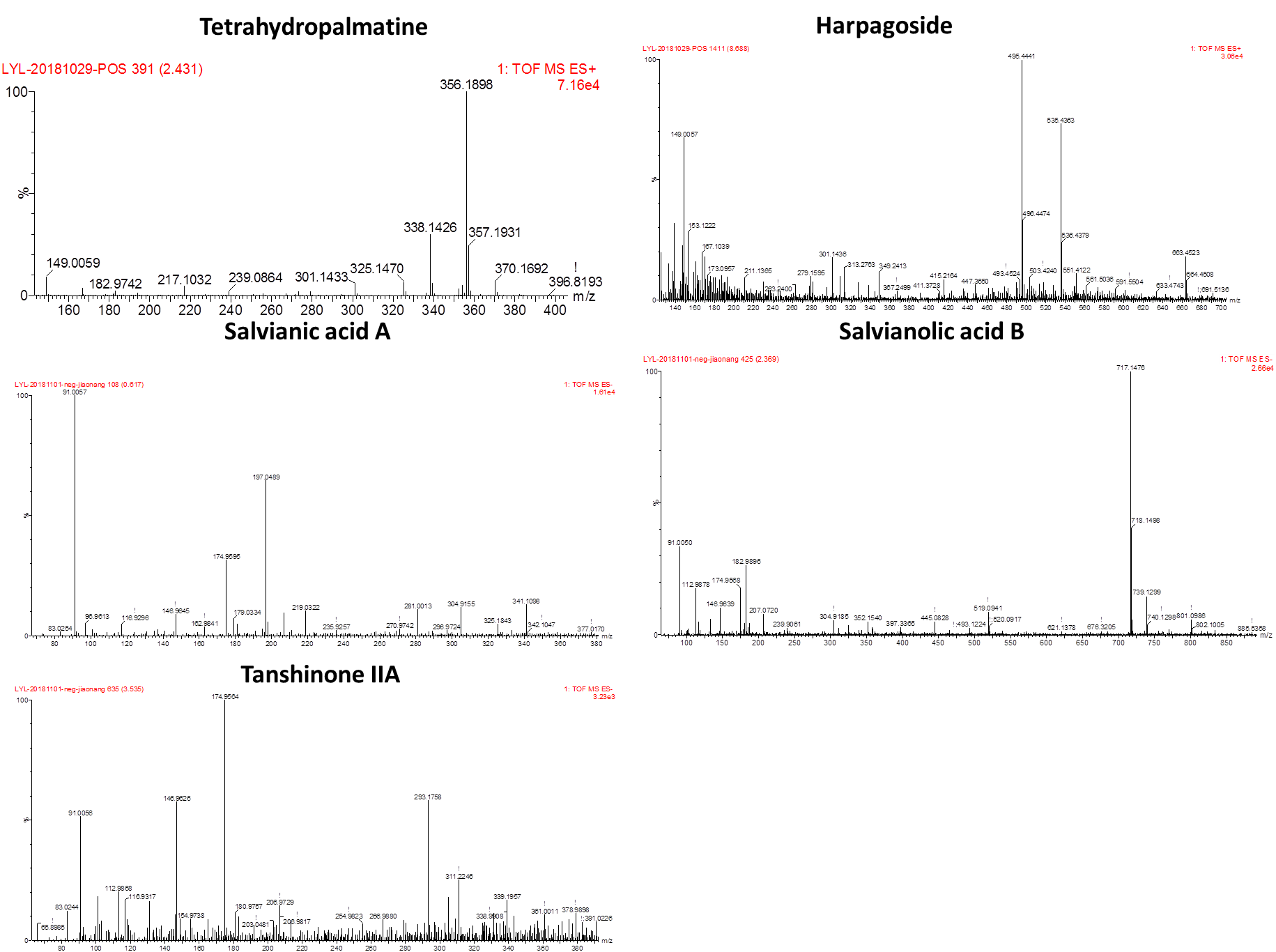

Supplement: Supplementary file 1 [file DataSheet_1.zip › Supplementary figures/Supplementary Figure 2.tif]

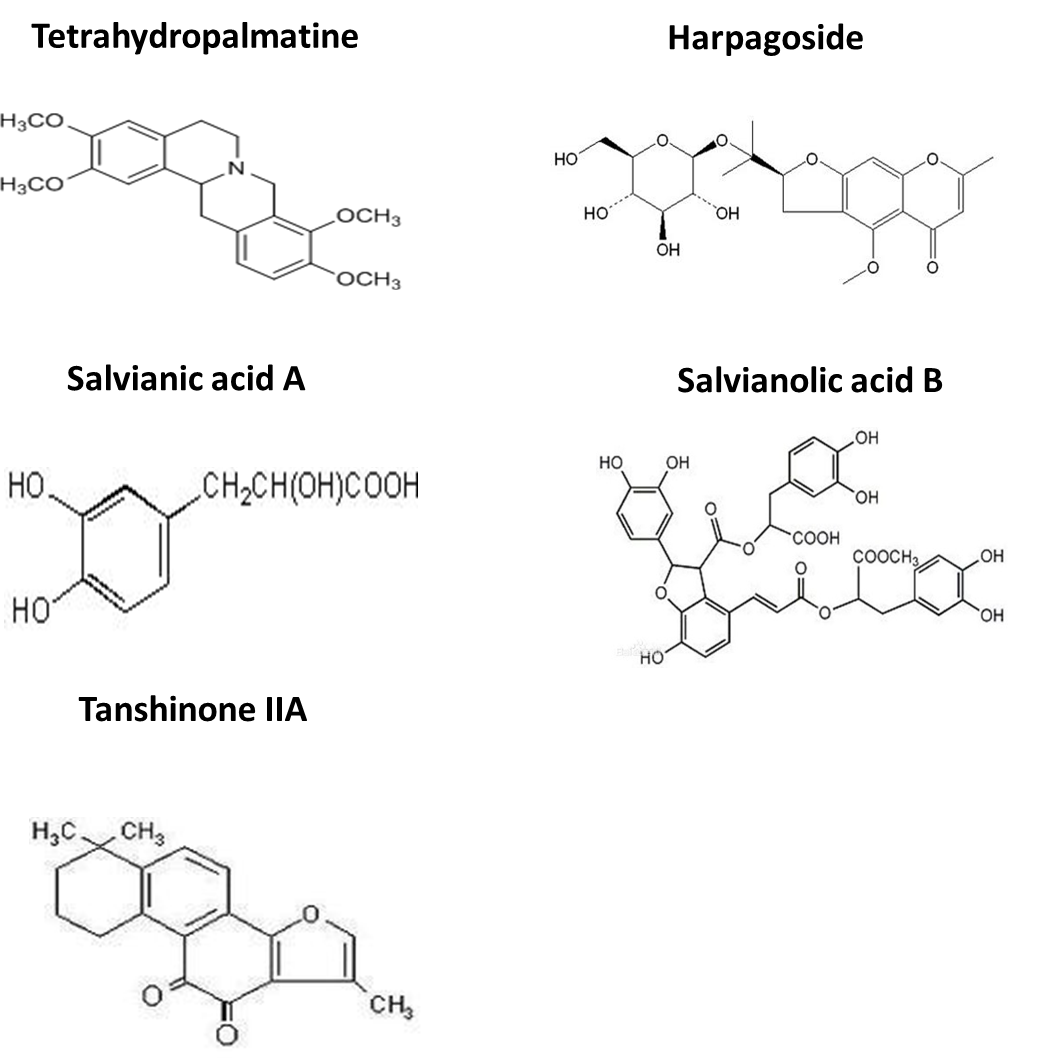

Supplement: Supplementary file 1 [file DataSheet_1.zip › Supplementary figures/Supplementary Figure 3.tif]
