# Supplementary material for: Shen-Yuan-Dan Capsule Attenuates Atherosclerosis and Foam Cell Formation by Enhancing Autophagy and Inhibiting the PI3K/Akt/mTORC1 Signaling Pathway
Source: Front Pharmacol. 2019 May 31;10:603. doi: 10.3389/fphar.2019.00603 (PMC6554665; doi:10.3389/fphar.2019.00603)
Supplement: Supplementary file 2 [file DataSheet_2.docx]

**Supplementary Figure legends**

Supplementary figure 1. Identification of major components of SYDC. The sample of SYDC was examined using UPLC–MS/MS. Data were collected and proceeded by software Masslynx 4.1. The positive (A) and negative (B) ion chromatograms of SYDC were shown as indicated.

Supplementary figure 2. The mass spectrograms of the main ingredients of SYDC.

Supplementary figure 3. The [chemical formula](http://www.baidu.com/link?url=xsbkQMjvT176N8daRsarLsQD-yEdVjbi0VSu2K-mXoM10_iEmrHdWwFiF22FMUmbVQ1n-CewQQCe36VFiFwEovBA2q2r9dVYakqm78d7bAnGzToSJRAU2zJkrNGG1oMS)s of the main ingredients of SYDC.
